# Ovatodiolide Suppresses Oral Cancer Malignancy by Down-Regulating Exosomal Mir-21/STAT3/β-Catenin Cargo and Preventing Oncogenic Transformation of Normal Gingival Fibroblasts

**DOI:** 10.3390/cancers12010056

**Published:** 2019-12-24

**Authors:** Jia-Hong Chen, Alexander T. H. Wu, Oluwaseun Adebayo Bamodu, Vijesh Kumar Yadav, Tsu-Yi Chao, Yew-Min Tzeng, Debabrata Mukhopadhyay, Michael Hsiao, Jih-Chin Lee

**Affiliations:** 1 Graduate and Institute of Clinical Medicine, College of Medicine, Taipei Medical University, Taipei 11031, Taiwan; ndmc_tw.tw@yahoo.com.tw; 2 Division of Hematology/Oncology, Department of Medicine, Tri-Service General Hospital, National Defense Medical Center, Taipei 114, Taiwan; 3 The PhD Program for Translational Medicine, College of Medical Science and Technology, Taipei Medical University, Taipei 11031, Taiwan; chaw1211@tmu.edu.tw; 4 Graduate Institute of Medical Sciences, National Defense Medical Center, Taipei 114, Taiwan; 5 Department of Hematology and Oncology, Cancer Center, Taipei Medical University-Shuang Ho Hospital, New Taipei City 23561, Taiwan; 16625@s.tmu.edu.tw (O.A.B.); j0607@ms6.hinet.net (T.-Y.C.); 6 Department of Medical Research & Education, Taipei Medical University-Shuang Ho Hospital, New Taipei City 23561, Taiwan; 7 The Program for Translational Medicine, Graduate Institute of Biomedical Informatics, College of Medical Science and Technology, Taipei Medical University, Taipei 11031, Taiwan; vijeshp2@gmail.com; 8 Graduate Institute of Biomedical Informatics, Taipei Medical University, Taipei 11031, Taiwan; 9 Taipei Cancer Center, Taipei Medical University, Taipei 11031, Taiwan; 10 Center for General Education, National Taitung University, Taitung 95092, Taiwan; president@nttu.edu.tw; 11 Department of Life Science, National Taitung University, Taitung 95092, Taiwan; 12 Department of Biochemistry and Molecular Biology, Mayo Clinic, Rochester, MN 55905, USA; Mukhopadhyay.Debabrata@mayo.edu; 13 Genomics Research Center, Academia Sinica, Taipei City 11529, Taiwan; mhsiao@gate.sinica.edu.tw; 14 Department of Biochemistry, Kaohsiung Medical University, Kaohsiung 80708, Taiwan; 15 Department of Otolaryngology, Head and Neck Surgery Tri-Service General Hospital, National Defense Medical Center, Taipei 114, Taiwan; 16 Department of Biological Science and Technology, Institute of Bioinformatics and Systems Biology, National Chiao Tung University, Hsinchu 300, Taiwan

**Keywords:** oral squamous cell carcinoma, ovatodiolide, extracellular vesicles, tumor microenvironment, cancer-associated fibroblasts, miR-21-5p/STAT3/β-catenin signaling

## Abstract

Oral squamous cell carcinoma (OSCC) is among the most commonly diagnosed malignancies in the world. Patients with OSCC often develop treatment resistance, resulting in a poor prognosis. Mounting evidence indicates that interactions between cancerous cells and other components of the tumor microenvironment (TME) determine their response to treatment. Herein, we examined the role of cancer stem cell-derived extracellular vesicles (CSC_EVs) generated from CAL27 and SCC-15 OSCC cells in the development of cisplatin (CDDP) resistance. We demonstrated that CSC_EVs enhance CDDP resistance, clonogenicity, and the tumorsphere formation potential of OSCC cells. Our bioinformatics analyses revealed that OSCC_EVs are enriched with microRNA (miR)-21-5p and are associated with increased metastasis, stemness, chemoresistance, and poor survival in patients with OSCC. Mechanistically, enhanced activity of CSC_EVs was positively correlated with upregulated β-catenin, phosphatidylinositol-3 kinase (PI3K), signal transducer and activator of transcription 3 (STAT3), mammalian target of rapamycin (mTOR), and transforming growth factor (TGF)-β1 messenger (m)RNA and protein expression levels. CSC_EVs also conferred a cancer-associated fibroblast (CAF) phenotype on normal gingival fibroblasts (NGFs), with the resultant CAFs enhancing the oncogenicity of OSCC cells. Interestingly, treatment with ovatodiolide (OV), the bioactive component of *Anisomeles indica*, suppressed OSCC tumorigenesis by reducing the cargo content of EVs derived from CSCs, suppressing self-renewal, and inhibiting the NGF-CAF transformation by disrupting EV-TME interactions. Moreover, by suppressing miR-21-5p, STAT3, and mTOR expressions in CSC_EVs, OV re-sensitized CSCs to CDDP and suppressed OSCC tumorigenesis. In vivo, treatment with OV alone or in combination with CDDP significantly reduced the tumor sphere-forming ability and decreased EV cargos containing mTOR, PI3K, STAT3, β-catenin, and miR-21-5p. In summary, our findings provide further strong evidence of OV’s therapeutic effect in OSCC.

## 1. Introduction

Despite advances in therapeutic agents for treating oral squamous cell carcinoma (OSCC), overall survival rates, functional outcomes, and treatment-associated toxicities remain suboptimal. The development of resistance against treatments is one of the most challenging tasks for managing OSCC and important obstacles in therapeutic development. Therefore, alternative approaches must be taken to tackle these issues in order to provide tools to combat malignant OSCC.

Accumulating evidence indicates that just as the presence of cancer stem cells (CSCs) contributes to tumor initiation, progression, and development of resistance [1,2], so also does the tumor microenvironment (TME) where tumor cells and diverse stromal cells cohabitate, facilitating a favourable niche for promoting tumorigenesis [3,4]. There is evidence that stromal cells, such as cancer-associated fibroblasts (CAFs), promote and maintain the generation of CSCs [5]; however, regardless of whether the reverse is possible, the probable underlying mechanism remains unclear, thus necessitating the unraveling of complex cellular interactions and signaling cascades between CSCs and stromal cells, thereby enabling the discovery of druggable targets and/or signaling pathways.

Exosomes are a small type of extracellular vesicle (EV), 30~100 nm of size, endocytic in origin [6], naturally released by all cell types, including cancer cells, and which mediate intercellular communication. Cancer EVs contain signaling proteins, lipids, and nucleic acids, including non-coding RNAs, that can reprogram recipient cells. Thus, coupled with their complicity in major steps of disease progression [7], EVs have attracted much attention in the field of cancer biology, with reports that cancer EVs contribute to drug resistance, TME reprogramming, and metastasis. Based on these premises, a better understanding of the functional complexities of EVs in tumorigenesis is essential for the development of novel therapeutic strategies that target exosome oncogenicity.

Ovatodiolide (OV) is the bioactive component of the medicinal herb *Anisomeles indica* (L.) Kuntze (Labiatae) with documented anti-inflammatory properties. Accumulating evidence including from our previous studies indicates that OV elicits anticancer effects by suppressing oncogenic markers such as tumor necrosis factor (TNF)-α, nuclear factor (NF)-κB, matrix metalloproteinases (MMPs), and signaling networks such as the Wnt/β-catenin, Hippo/YAP1, and phosphatidylinositol-3 kinase (PI3K)/mammalian target of rapamycin (mTOR) pathways [8,9,10]. Since activation of these signaling networks is characteristic of both cancer cells and stromal cells within the TME, we examined whether OV-mediated anticancer activities could be exploited to disrupt intra- and intercellular communications within the TME.

In this study, we explored the role of CSCs in the development of chemotherapeutic (cisplatin, CDDP) resistance in OSCC. Our review of the contemporary literature revealed that signaling molecules, including oncogenic microRNA (miR)-21-5p, components of the PI3K/mTOR/signal transducer and activator of transcription 3 (STAT3) signaling cascade, and transforming growth factor (TGF)-β1 were found in EVs derived from CSCs (CSC_EVs) [11,12,13,14]. Coculture of the SCC-15 and CAL27 parental OSCC cell lines with CSC_EVs was shown to promote malignant OSCC phenotypes, including enhanced migration, invasion, self-renewal, and CDDP resistance. Additionally, CSC_EVs facilitated the transformation of normal gingival fibroblasts (NGFs) into cancer-associated fibroblasts (CAFs), suggesting CSC-EVs’ capacity for TME-reprogramming. Subsequently, OV treatment significantly suppressed the oncogenic potential of CSC_EVs by reducing their cargo content. An in vivo study also demonstrated the efficacy of OV in inhibiting OSCC tumorigenesis using a CDDP-resistant tumor xenograft mice model after inoculation of CDDP-resistant CAL27 tumorsphere cells. Interestingly, the combination of OV and CDDP effectively suppressed tumor growth and significantly improved the survival rate of treated mice, compared to control mice. Notably, OV-mediated effects were associated with reduced stemness and depleted PI3K, mTOR, STAT3, and miR-21-5p cargo contents of CAL27-derived EVs.

In summary, we provide preclinical evidence that OV treatment suppresses tumorigenesis and OSCC stemness, as well as normalizes the TME, by reducing the oncogenic cargo in CSC-EVs. Thus, OV is a potential adjuvant anticancer therapeutic agent for treating patients with chemoresistant OSCC.

## 2. Materials and Methods

### 2.1. Cell Culture and Reagents

Human OSCC cell lines, SCC-15 and CAL27, and primary NGFs (PCS-201-018) were obtained from American Type Culture Collection (Manassas, VA, USA) and were maintained according to culture methods recommended by the vendor. Pure OV crystals (purity 99.7%, MW 328.4) were provided by Prof. Yew-Min Tzeng (National Taitung University, Taitung, Taiwan); the crystals were ground up and dissolved in dimethyl sulfoxide (DMSO) as a stock solution (100 mM) and stored at −20 °C in the dark until further use. Cisplatin (CDDP) was purchased from Selleckchem (cat no. S1166, Hsinchu County, Taiwan).

### 2.2. Bioinformatics Search

Exosomal microRNA profiling (heatmap) from six patients with OSCC were obtained and analyzed from a previous study [7]. The negative correlation between miR-21 and its target PCDC4 (a major tumor suppressor) was computed using the ECORI online tool (http://starbase.sysu.edu.cn/). The analysis of PIK3CA and STAT3 expressions and their associations with the survival ratio in a head and neck cancer patient cohort (GSE26549) were analyzed using SurvExpress software. The graphs were re-organized for better presentation.

### 2.3. Tumor Sphere Generation

Tumor spheres of SCC-15 and CAL27 cells were generated under serum-deprived culture conditions according to a previously established protocol [15] with slight modifications. In brief, OSCC cells were seeded (5000 cells/well) in six-well ultra-low-attachment plates (Corning, Corning, NY) in Dulbecco’s modified Eagle medium (DMEM)/F12 medium supplemented with B27 and 20 ng/mL basic fibroblast growth factor (bFGF) (Invitrogen, Carlsbad, CA, USA) and epidermal growth factor (EGF) (20 ng/mL, Millipore, Bedford, MA). Cells were allowed to aggregate and grow for at least 7 days, and cell aggregates (diameter >50 µm) were considered a tumor sphere and counted with an inverted phase-contrast microscope. STAT3 was silenced in both CAL27 and SCC-15 cells using the siRNA technique (siRNA ID, s743; nc, negative control, cat#4390843, ThermoFisher Scientifics, Taipei, Taiwan). The transfection experiments were carried out according to the protocols provided by the vendor with slight modifications. The amount of silencer (siRNA) used in our study was 2× concentrations suggested by the original protocol. 

### 2.4. Cell Viability Assay

A sulforhodamine B (SRB) assay was performed to determine the efficacy of CDDP and OV. Briefly, OSCC cells (5000 cells/well) were seeded in 96-well plates and treated with different concentrations of CDDP and OV for 48 h. Post-treatment, cells were washed and fixed with trichloroacetic acid (TCA; 10%) and incubated at 4 °C for 1 h. Subsequently, cells were washed three times with double-distilled (dd) H_2_O and air-dried. Cells in the dried plates were subjected to a 10-min incubation with 100 µL of 0.4% (*w*/*v*) SRB (prepared in 1% (*v*/*v*) acetic acid). Any unbound dye was removed by washing with 1% acetic acid. Stained cells were briefly incubated in 20 mmol/L Tris base on a shaker. Optical densities were measured with a microplate reader (Molecular Devices, Sunnyvale, CA, USA) at 562 nm.

### 2.5. Isolation of EVs

EVs (or exosomes) were isolated from culture media using the Total Exosome Isolation Reagent (Thermo Fisher Scientific, Taipei, Taiwan). The protocol was carried out according to the vendor’s instruction manual. Cluster of differentiation 9 (CD9) and CD63 antibodies were used to verify and semiquantitatively measure the amount of EVs isolated. According to our protocol, 15 mL of culture medium (from the CAL27 tumor sphere culture) on average yielded 900~1000 µg of total protein (isolated CSC_EVs), as determined using a Bradford protein assay.

### 2.6. Coculture Experiments

The CAL27 and SCC-15 OSCC cell lines were seeded at 5 × 10^5^ cells/mL into the upper chamber (insert with a membrane, 0.4-μM pore size, Corning, Lowell, MA), and NGF cells were seeded at 10^5^ cells/mL in the lower chamber. Cells were cultured in DMEM supplemented with 5% fetal bovine serum (FBS) for 96 h. In the case of CSC_EV coculture, CAL27 and SCC-15 cells (5 × 10^5^ cells/well) were seeded in six-well plates and 100 µL of total CSC_EVs (approximately 900 µg, isolated from 15 mL of serum-deprived medium of CAL27 and SCC-15 CSCs) was added and cultured for 48 h. Cocultured cells (i.e., CAL27 + CSC_EVs and SCC-15 + CSC_EVs or NGFs + EVs and NGFs + CSC_EVs) were subsequently harvested or further maintained for additional analyses.

### 2.7. Immunofluorescence Imaging

NGFs and thereafter transformed CAFs were plated in six-well chamber slides (Nunc™, Thermo Fisher Scientific, Rochester, NY, USA) for 24 h. An immunofluorescence experiment was carried out using a previously established protocol according to the vendor’s instructions. Primary antibodies were then added and incubated at room temperature for 1 h. The primary antibodies used were α-smooth muscle actin (α-SMA, 1:100, cat no. 48938) and vimentin (Vim, cat no. 5741, 1:100, Cell Signaling Technology, Danvers, MA, USA). Matched secondary antibodies were anti-mouse immunoglobulin G (IgG) (H+L), F(ab)2 fragment (1:800, AlexaFluor 488 conjugate, cat no. 4408) and anti-rabbit IgG (1:600, AlexaFluor 555 conjugated, cat no. 4413). Stained cells were mounted using Vectashield mounting medium with 4′,6-diamidino-2-phenylindole (DAPI) to counterstain the DNA. Cells were imaged on a Zeiss Axiophot (Carl Zeiss, Germany) fluorescence microscope. Microphotographs were captured using an AxioCam MRc digital video camera and analyzed using AxioVision Zeiss software (Carl Zeiss).

### 2.8. Sodium Dodecylsulfate Polyacrylamide Gel Electrophoresis (SDS-PAGE) and Western Blotting

Total protein lysates from OSCC cell lines (parental, tumor spheres, and those from coculture experiments) were harvested using a lysis buffer composed of proteinase inhibitors. After centrifugation, 40 μg of the protein lysate in loading buffer was loaded into each lane. Protein samples were separated using standard SDS-PAGE procedures (Bio-Rad, Hercules, CA, USA) and transferred onto a polyvinylidene difluoride membrane. Membranes were then blocked and incubated with primary antibodies. Details of antibodies along with dilutions used for this study are listed in the Supplementary Table S1.

### 2.9. Real-Time Polymerase Chain Reaction (PCR)

Total RNA was isolated and purified using a TRIzol-based protocol (Life Technologies) according to the vendor’s instructions. Total RNA (500 ng) was reverse-transcribed (RT) using a Qiagen OneStep RT-PCR Kit (Qiagen, Taiwan), and the PCR was performed using a Rotor-Gene SYBR Green PCR Kit (400, Qiagen). Primers sequences for the quantitative (q)PCR experiments are listed in Table S2 in Supplementary Materials.

### 2.10. Migration and Invasion Assay

A transwell system was used to assess both the migratory and invasive abilities of OSCC cells and transformed CAFs. In brief, NGFs (2 × 10^4^ cells/well) or CAFs (transformed by pre-incubating with EVs or CSC_EVs, 900 µg, 48 h) were seeded into the upper chambers which contained 200 μL serum-free DMEM, and 500 μL DMEM with 10% FBS in the lower chambers to create a gradient. The cells were incubated for 16–24 h, and then the membranes were fixed with formaldehyde (10%) followed by crystal violet staining. Cells on the upper side of the membrane were removed and cells that had invaded the opposite of the membranes were counted. With regards the invasion assay, the membrane in the upper chamber was pre-coated with Matrigel (BD Bioscience).

### 2.11. Colony Formation Assay

The colony-forming ability of OSSC cells with and without treatments was assessed as per a previously described protocol from Franken et al. [16]. Briefly, 500 OSSC cells were plated in six-well plates (Corning) and treated with OV. Plates were incubated, and cells were allowed to grow for another 7 days and then were fixed, stained, and counted.

### 2.12. In Vivo Evaluation of the Therapeutic Potential of OV

All animal experiments performed in this study were approved by the Institutional Animal Care and Use of Committee or Panel (IACUC/IACUP) of Taipei Medical University (approval no.: LAC-2018-0414). Immune-compromised NOD/SCID mice (6~8 week old, females, BioLASCO, Taipei, Taiwan) were injected with CAL27 CSCs (tumor spheres generated under serum-deprived conditions, at 10^6^ cells/injection) subcutaneously to establish the CDDP-resistant xenograft mouse model. Mice were allowed to recover and develop tumors. When the tumor size became palpable, mice were randomly divided into four groups: a vehicle control, CDDP-only (5 mg/kg, i.p. injection, once a week), OV-only (5 mg/kg, i.p. injection, five times/week), and their combination (CDDP + OV). The tumor volume and body weight (BW) were monitored and measured on a weekly basis. Mice were humanely sacrificed via a cervical dislocation method at the end of the experimental period. Tumor samples were resected for further analyses. The tumor volume was calculated as the tumor size = length × width^2^/2.

### 2.13. Statistical Analysis

All experiments were performed at least three times. Experimental data are presented as the mean ± standard deviation (SD). An analysis of variance (ANOVA) was used to evaluate the statistical significance of mean values. The Kaplan-Meier method was used for the survival analysis of the animal experiment. A value of *p* < 0.05 was considered statistically significant.

## 3. Results

### 3.1. EVs from CSCs Promote the Malignant Phenotype of OSCC Cells by Enhancing PI3K/mTOR/STAT3 Signaling

**Figure 1:**
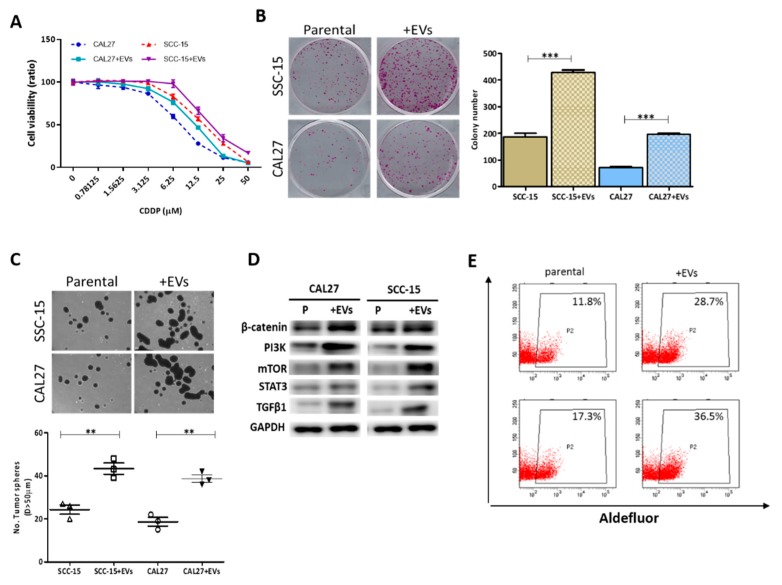
Extracellular vesicles (EVs) from cancer stem cells (CSCs) promoted malignant properties of oral squamous cell carcinoma (OSCC) cells. (**A**) A cell viability assay indicated that CSC_EV-cocultured CAL27 and SCC-15 cells showed markedly increased resistance against cisplatin (CDDP) compared to parental cells without CSC_EVs. (**B**) Increased colony-forming ability of both CAL27 and SCC-15 cells cocultured with CSC_EVs. (**C**) Increased migratory capacity of CAL27 and SCC-15 cells cocultured with CSC_EVs. (**D**) Western blot analysis showing that CSC_EVs promoted the stemness marker, β-catenin, and the oncogenic markers, phosphatidylinositol-3 kinase (PI3K), signal transducer and activator of transcription 3 (STAT3), mammalian target of rapamycin (mTOR), and transforming growth factor (TGF)-β1in parental CAL27 and SCC-15 cells. (**E**) Flow cytometric analysis showing that CSC_EVs’ coculture significantly increased aldehyde dehydrogenase 1 (ALDH1) activity within both CAL27 and SCC-15 cells. * *p* < 0.05; ** *p* < 0.01; *** *p* < 0.001.

To explore the tumor-promoting function of CSC-EVs, we isolated EVs from CAL27 and SCC-15 tumorspheres cultured in serum-deprived culture media. We demonstrated that coculturing parental CAL27 and SCC-15 cells with CSC_EVs enhanced cell viability (Figure 1A) suggesting a role for CSC_EVs in the markedly increased CDDP resistance of cocultured CAL27 or SCC-15 cells, compared to their parental counterparts. We also showed that when cocultured with CSC-EVs, the ability of CAL27 or SCC-15 cells to form colonies was significantly enhanced by 2.67- (*p* < 0.001) and 2.24-fold (*p* < 0.001), respectively (Figure 1B). In addition, the tumorsphere-formation capability was increased by 2.04- (*p* < 0.01) and 1.76-fold (*p* < 0.01), respectively, in cocultured CAL27 and SCC-15 cells, compared to parental cells (Figure 1C). We also observed upregulated β-catenin, PI3K, mTOR, STAT3, and TGFβ1 protein expression levels in CSC-EVs cocultured with OSCC cells, compared to their parental counterparts (Figure 1D). Moreover, CSC-EVs enhanced the aldehyde dehydrogenase 1 (ALDH1) activity of CAL27 and SCC-15 cells by 16.9% and 19.2%, respectively (Figure 1F).

### 3.2. EVs from CSCs Transport Oncogenic Signaling Molecules

**Figure 2:**
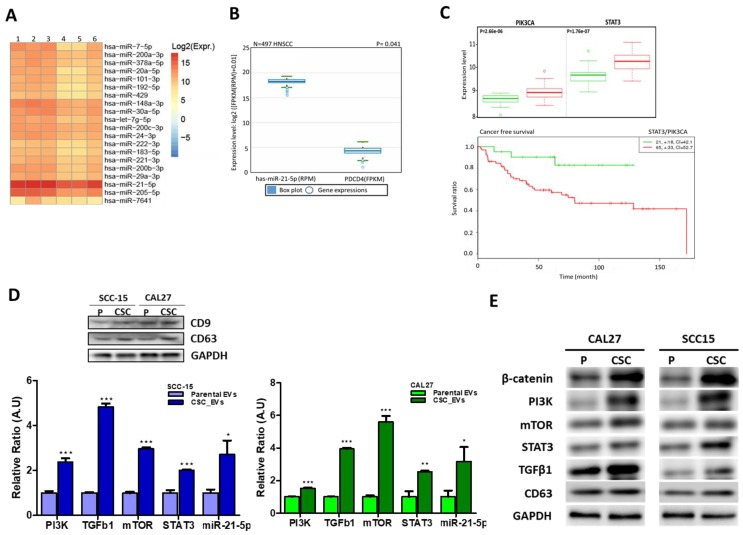
Cancer stem cells (CSCs) secrete extracellular vesicles (CSC_EVs) with oncogenic potential. (**A**) miR-21 is abundantly contained within EVs collected from six clinical oral squamous cell carcinoma (OSCC) samples [13]. (**B**) TCGA database analysis showed a strong reverse association between miR-21 and its target, the PDCD4 tumor suppressor. (**C**) Analysis of the GSE26549 database showed that increased PIK3CA and signal transducer and activator of transcription 3 (STAT3) mRNA levels were strongly associated with a higher risk of OSCC (upper panel) and shorter survival (lower panel, log-rank equal curves *p* = 0.00148). A comparative qPCR analysis (**D**) and Western blots (**E**) between cargo contents of CSC_EVs and EVs (parental cells). The insert depicts Western blots of cluster of differentiation 9 (CD9) and CD63 (markers of EVs) isolated from parental and tumor spheres (CSCs) of CAL27 and SCC-15 cells. CSC_EVs contained significantly higher levels of phosphatidylinositol-3 kinase (PI3K), transforming growth factor (TGF)-β1, mammalian target of rapamycin (mTOR), STAT3, and miR-21-5p compared to the parental EV counterparts. * *p* < 0.05; ** *p* < 0.01; *** *p* < 0.001.

Next, using a bioinformatics analysis of a public database [13], we performed miRNA profiling of EVs secreted by oral cancer cells to identify potential candidate oncogenic microRNAs (oncomiRs) in EVs. We observed that miR-21 was the most abundant exosomal miR in all six OSCC samples sequenced (Figure 2A). It is well documented that miR-21 targets tumor suppressors such as PDCD4 and phosphatase and tensin homolog (PTEN) [17], both of which suppress expressions of PI3K, STAT3, mTOR, and β-catenin [18]. In addition, after a re-analysis of The Cancer Genomic Atlas head and neck squamous cell carcinoma (TCGA-HNSCC) (*n* = 497) (https://www.cancer.gov/tcga), we demonstrated elevated miR-21-5p levels in patients with HNSCC, and this was inversely correlated with expression of the PDCD4 tumor suppressor (Figure 2B). Similarly, our analysis of the GSE26549 database of gene profiles of ‘patients with oral preneoplastic lesions (OPLs) having high risk of developing oral cancer’ [19], revealed that increases in PI3K (*p* = 2.66 × 10^−6^) and STAT3 (*p* = 1.76 × 10^−7^) gene expressions were associated with an increased risk of oral cancer (Figure 2C, upper panel), with high PI3K/STAT3 dual-gene expression associated with worse disease-free survival (DFS) (Figure 2C, lower panel). Previous reports indicated that EVs secreted from cancer cells transport transcripts and/or proteins with oncogenic properties [7,20,21]. We then isolated CD9^high^CD63^high^ EVs secreted by CAL27- or SCC-15-derived CSCs. Our quantitative polymerase chain reaction (qPCR) analysis showed that these CSC_EVs exhibited significantly higher expression levels of PI3K, mTOR, STAT3, and TGFβ1 mRNAs, as well as miR-21-5p, compared to the EVs secreted from the parental counterparts (Figure 2D). To investigate the oncogenic and stemness promoting roles of STAT3 in OSCC cells, we applied small interfering RNA (siRNA) to knockdown STAT3 expression in CAL27- or SCC-15 cells. These STAT3-silenced cells were further used for experiments (Supplementry Figure S1). STAT3 knockdown resulted in the reduction of OSCC tumorigenesis/stemness (decrease in the OSCC tumor sphere formation) and oncogenic exosomal cargo contents such as β-catenin, TGFβ1 and miR-21-5p. Reduction in migration, as well as sensitization of OSSC CSCs to CDDP, is described in the Supplementary Figure S1. 

### 3.3. CSC_EVs Promote the Transformation of Human NGFs into CAFs

**Figure 3:**
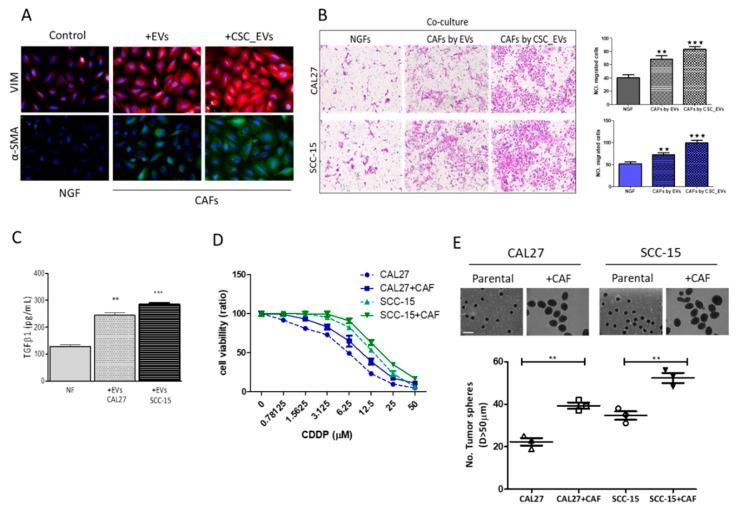
Extracellular vesicles (EVs) from cancer stem cells (CSCs) promoted cancer-associate fibroblast (CAF) transformation and increased oral squamous cell carcinoma (OSCC) stemness. (**A**) Immunofluorescence imaging showed increased expressions of both α-smooth muscle actin (α-SMA) and vimentin (Vim) in normal gingival fibroblasts (NGFs) after coculturing with CSC_EVs. Middle lane, CSC_EVs from CAL27 cells; right lane, CSC_EVs from SCC-15 cells. (**B**) Increased migratory ability in CAL27 and SCC-15 cells co-cutlured with CAFs by EVs (EVs from parental cancer cells) and by CAFs; by CSC_EVs (EVs generated from respective CSCs), as compared to their NGF counterparts. (**C**) An ELISA (enzyme-linked immunosorbent assay) showed that CSC-EV-transformed CAFs secreted a significantly greater amount of transforming growth factor (TGF)-β1 into the culture medium. (**D**,**E**) CAFs transformed by CSC_EVs enhanced cisplatin (CDDP) resistance and tumor sphere generation in both CAL27 and SCC-15 cells. +CAF, co-cultured with CAF. * *p* < 0.05; ** *p* < 0.01; *** *p* < 0.001.

CAFs were shown to induce and/or facilitate the progression of many cancer types, including OSCC [3]. Here, we showed that CAL27 and SCC-15 CSC-EVs induced the transformation of NGFs into CAFs. We demonstrated that NGFs cocultured with CSC_EVs expressed markedly higher levels of CAF markers, namely α-smooth muscle actin (α-SMA) and vimentin (Vim) (Figure 3A). In addition, the resultant CAFs exhibited significant increases in migratory and invasive capabilities (Figure 3B) compared to their NGF counterparts. More importantly, CSC_EV-transformed NGFs secreted a substantially higher amount of TGF-β1, a key CAF cytokine, into the culture medium (Figure 3C). Furthermore, the transformation of NGFs into CAFS by CSC_EVs conferred a CDDP-resistant phenotype on cocultured CAL27 and SCC-15 cells (Figure 3D). Consistently, after coculturing with NGF-derived CAFs, we observed an increased ability to generate tumorspheres by CAL27 (1.90-fold, *p* < 0.01) and SCC-15 (1.45-fold, *p* < 0.01) cells compared to parental cells (Figure 3E).

### 3.4. OV Suppresses OSCC Oncogenicity by Reducing Exosomal Cargos

**Figure 4:**
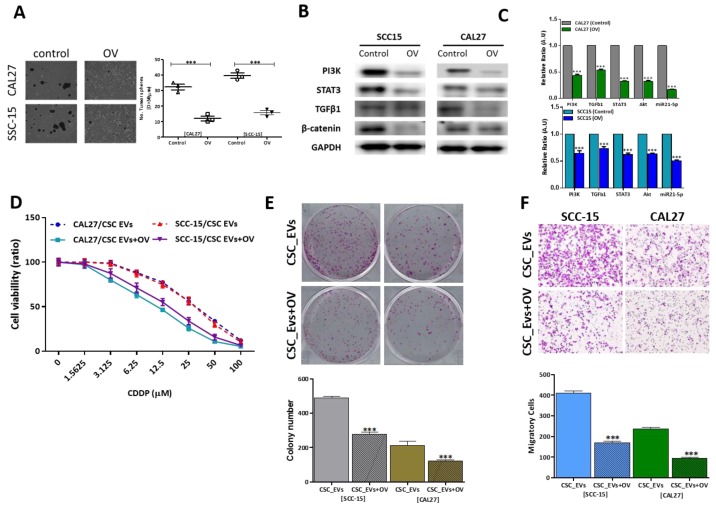
Ovatodiolide (OV) treatment suppresses tumorigenesis via reduction of the exosomal cargo. (**A**) OV treatment (4 µM, 48 h) significantly reduced the number of tumor spheres generated in both the CAL27 and SCC-15 cell lines. (**B**) Western blots show that OV treatment suppressed phosphatidylinositol-3 kinase (PI3K), signal transducer and activator of transcription 3 (STAT3), transforming growth factor (TGF)-β1, and β-catenin in tumor spheres generated after OV treatment. (**C**) OV treatment led to significantly reduced mRNA levels of PI3K, TGF-β1, Akt, STAT3, and miR-21-5p in cancer stem cell (CSC)_extracellular vesicles (EVs), compared to their control counterparts. a, *p* < 0.01; b, *p* < 0.001. CSC_EVs isolated after OV treatment showed reduced cisplatin (CDDP) resistance (**D**), and significantly suppressed colony-forming (**E**) and migratory (**F**) abilities. ** *p* < 0.01; *** *p* < 0.001.

In addition to the demonstrated ability of OV to inhibit carcinogenesis in different cancer types by our group [8,9,10,22], the present study demonstrated that treatment with OV significantly suppressed the ability of CAL27 (2.91-fold, *p* < 0.001) and SCC-15 (2.67-fold, *p* < 0.001) cells to form tumorspheres (Figure 4A) with concomitant downregulation of PI3K, STAT3, TGFβ1, and β-catenin protein expression levels in OV-treated tumorspheres, compared to their control counterparts (Figure 4B). Next, our comparative analysis of gene expression profiles of CSC_EVs from control and OV-treated groups showed that CSC_EVs from OV-treated cells contained significantly lower amounts of cargo loads of PI3K, TGF-β1, STAT3, AKT, and miR-21-5p, compared to control CSC_EVs (Figure 4C). In addition, we demonstrated that 4 µM OV significantly enhanced the ability of cisplatin to suppress the viability of CSC_EV-cocultured CAL27 and SCC-15 cells, compared to their untreated counterparts (Figure 4D). Treatment with 4 µM OV reduced the number of colonies formed by treated SCC-15 (1.78-fold, *p* < 0.001) and CAL27 (1.82-fold, *p* < 0.001) cells, compared to the untreated group (Figure 4E). Similarly, we demonstrated that when treated with 4 µM OV, CSC_EV-cocultured SCC-15 and CAL27 cells lost their ability to migrate by 2.35- (*p* < 0.001) and 2.3-fold (*p* < 0.001), respectively (Figure 4F).

### 3.5. OV Treatment Suppresses the Transformation of NGFs into CAFs

**Figure 5:**
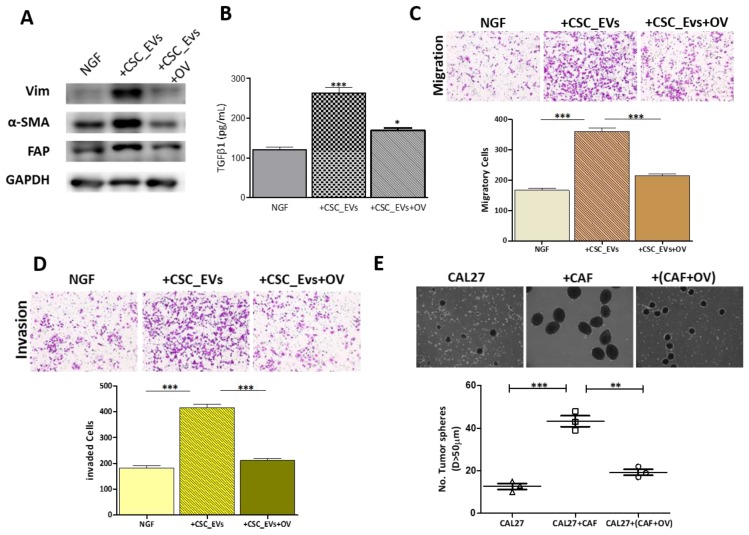
Ovatodiolide (OV) reduced cancer-associated fibroblast (CAF) transformation. (**A**) Western blots showed decreased levels of the CAF markers, fibronectin attachment protein (FAP), vimentin (Vim), and α-smooth muscle actin (α-SMA) in CAFs generated from cancer stem cell (CSC)_extracellular vesicles (EVs)+OV compared to ones cocultured with CSC_EVs. (**B**) The ELISA analysis showed that CAFs generated from normal gingival fibroblasts (NGFs) cocultured with CSC_EVs+OV secreted a significantly lower amount of transforming growth factor (TGF)-β1, compared to CAFs transformed from NGFs cocultured with CSC_EVs. (**C**,**D**) Migration and invasion assays demonstrated that CAFs generated from NGFs cocultured with CSC_EVs+OV had significantly lower migratory and invasive abilities, compared to their counterparts with CSC_EVs. (**E**) The tumor sphere formation assay indicated that CAL27 cells formed a significantly lower number of tumor spheres when cocultured with CAFs (CAFs+OV, transformed from NGFs, cocultured with CSC_EVs+OV). * *p* < 0.05; ** *p* < 0.01; *** *p* < 0.001.

Furthermore, we examined the ability of OV to affect the TME. Our results showed that compared to the untreated control, 4 µM OV downregulated the protein expressions of the CAF markers vimentin, α-SMA, and fibronectin attachment protein (FAP) in treated cells (Figure 5A), thus indicating that OV-treated CSC_EVs (CSC_EVs+OV) exhibited a significantly lower ability to transform NGFs into CAFs, compared to control CSC_EVs. In addition, the amount of TGF-β1 secreted by NGF-derived CAFs was significantly suppressed by exposure to 4 µM OV (1.53-fold, *p* < 0.05) (Figure 5B). Furthermore, OV-treated CAFs were significantly less migratory (1.71-fold, *p* < 0.001) (Figure 5C) and invasive (1.95-fold, *p* < 0.001) (Figure 5D). More importantly, OV-treated cocultured CAL27 cells were significantly less capable of forming tumorspheres compared to their untreated counterparts (Figure 5E).

### 3.6. OV Treatment Significantly Suppressed CDDP Resistance and Tumorigenesis in Vivo

**Figure 6:**
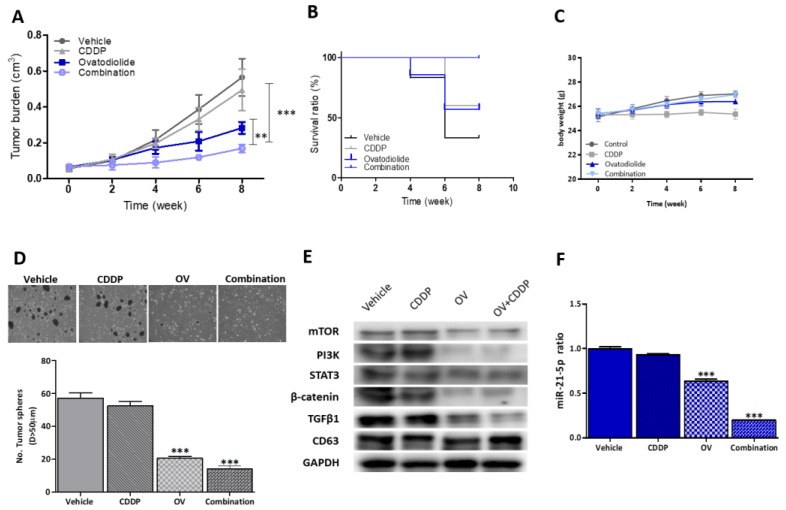
Ovatodiolide (OV) suppresses cisplatin (CDDP)-resistant CAL27 cancer stem cells (CSCs) in vivo. (**A**) Tumor volume versus time curve. The combination of OV+CDDP showed the most significant tumor suppressive effect followed by OV-only treatment, while groups treated with CDDP-only or the vehicle showed the largest tumor volumes. (**B**) Kaplan-Meier survival curve. Mice that received the combined OV+CDDP regimen showed the highest survival ratio followed by the OV-only group, while CDDP-only and vehicle mice showed the lowest survival ratios. (**C**) The body weight over time curve shows that all mice exhibited a steady increase in body weight over time, except for a slight decrease in mice which received the CDDP regimen. This suggests no apparent systemic toxicity of OV treatment. (**D**) Comparative tumor sphere-forming assay. Tumor samples collected from all experimental groups showed that OV+CDDP samples were the least capable of generating tumor spheres followed by the OV-only regimen, while the CDDP and vehicle groups were indifferent. (**E**) Western blots of extracellular vesicles (EVs) collected from all experimental groups. EVs from OV+CDDP and OV-only regimens showed a markedly lower level of mammalian target of rapamycin (mTOR), phosphatidylinositol-3 kinase (PI3K), and β-catenin compared to those from the vehicle and CDDP-only groups. Cluster of differentiation 63 (CD63) was used as a marker of EVs and a loading control. (**F**) The qPCR analysis showed the lowest level of miR-21-5p in EVs collected from the combined OV+CDDP regimen followed by OV-only, while there was no significant difference between the vehicle and CDDP-only groups. ** *p* < 0.01; *** *p* < 0.001.

Next, using a tumor xenograft mice model, we evaluated the inhibitory effects of OV in vivo by implanting CDDP-resistant CAL27 CSCs into NOD/SCID mice. We demonstrated that compared to vehicle-treated control mice with a median tumor burden of 0.58 cm^3^ by week 8 and those treated with CDDP only (0.46 cm^3^), mice treated with combined OV and CDDP exhibited the smallest tumor burden (0.18 cm^3^), followed by the OV-alone group (0.27 cm^3^) (Figure 6A). An analysis of treatment-related changes in mice body weights (BWs) showed that while there was no apparent difference in the median BWs of mice treated with the vehicle, OV-alone, or CDDP+OV, CDDP-treated mice showed a slight decline in their median BW (Figure 6C). Using a Kaplan-Meier survival curve, we demonstrated that OV-alone or CDDP+OV conferred a significant survival advantage on mice, compared to the CDDP-alone and vehicle-treated groups (Figure 6B). Ex vivo, the ability to form tumorspheres was profoundly suppressed in tumor samples harvested from CDDP+OV- (4.75-fold, *p* < 0.001) and OV-alone-treated (2.85-fold, *p* < 0.001) mice, compared to CDDP-alone-treated mice and the vehicle-treated control group (Figure 6D). Consistent with earlier findings, this suppression of the tumor burden and stemness in the OV-alone and CDDP+OV groups was associated with decreased expressions of mTOR, PI3K, STAT3, β-catenin, and TGF-β1 proteins in CSC_EVs generated from tumor samples harvested from OV-alone or CDDP+OV-treated mice, compared to the CDDP-alone- or vehicle-treated groups (Figure 5E). Similarly, the miR-21-5p expression level was significantly downregulated in the OV-alone- (40% decrease, *p* < 0.001) and CDDP+OV-treated (~79%, *p* < 0.001) groups, compared to the CDDP-alone- and vehicle-treated groups (Figure 6F).

## 4. Discussion

Acquired chemoresistance represents a major challenge in the management of patients with OSCC [23,24]. The presence of CSCs was demonstrated in many cancer types, including OSCC, and they have been implicated in the development of treatment resistance [25,26]. Accumulating evidence indicates that CSCs are endowed with an enhanced ability to self-renew, efflux chemotherapeutic agents, and increase the epithelial-to-mesenchymal transition (EMT), as well as facilitate the production of a broad spectrum of oncogenic factors that constitutively remodel the TME which favors cancer progression [27,28]. Therefore, vigorous efforts have been dedicated in developing CSC-targeted interventions.

The present study implicates CSCs in promoting OSCC, particularly the role of CSC_EVs in remodeling of the TME. Our data revealed that CSC_EVs increased CDDP resistance, metastasis, and the self-renewal capability of OSCC CAL27 and SCC-15 cells, with a concomitant increase in expressions of several oncogenic markers such as PI3K, STAT3, mTOR, TGF-β1, and the stemness marker β-catenin, in CAL27 and SCC-15 cells after their coculture with CSC_EVs. These findings are consistent with those of previous studies that demonstrated that enhanced PI3K/mTOR/STAT3 signaling promotes chemoresistance, stemness, and the metastatic potential of oral cancer cells [25,29].

We also demonstrated that miR-21 represents one of the most abundant miRs transported within EV cargos secreted by oral cancer cells and that CSC_EVs contained a significantly higher level of miR-21 than their parental counterparts. This is of translational relevance, considering that miR-21 is a well-established oncogenic miR whose major targets include the tumor suppressors PTEN and PDCD4 [30,31], both of which inhibit PI3K/AKT/mTOR signaling and the Wnt/β-catenin cascade [30,32]. Moreover, EV miR-21 was implicated in resistance of recipient cancer cells to anticancer therapeutics [12,33]; in agreement, our bioinformatics analysis showed that miR-21 was the most abundant miR encapsulated within salivary EVs from six patients with OSCC. Notably, we also provided evidence that PI3K, STAT3, mTOR, and TGF-β1 mRNA and protein expressions are upregulated in CSC_EVs, compared to EVs isolated from parental CAL27 and SCC-15 cells. These findings are concordant with those showing that EVs from cancer cells carry signaling molecules such as proteins, lipids, and nucleic acids can transform the phenotype and genotype of recipient cells [34,35]. 

Furthermore, our data also demonstrated that TGF-β1-containing CSC_EVs possibly participate in the transformation of NGFs into CAFs and might promote their metastatic potential, thereby transforming the TME. This is in part consistent with previous evidence that CAF-secreted EVs facilitate the tumorigenesis of oral cancer cells [36,37,38]. However, the observation that CSC_EVs promoted the transformation of NGFs to CAFs is unique to this study and is the first such documentation, to the best of our knowledge. Our findings provide additional insights into how CSCs facilitate oral cancer progression by transforming the TME. We thus posit that CAFs transformed by EVs from both parental CAL27 and SCC-15 cells (or their tumor spheres) significantly promote CDDP-resistance and increased migratory and tumorsphere-forming capability when co-cultured with CAL27 and SCC-15 cells. A recent study showed that α-SMA expression was detected in nearly 70% of tumors and was positively correlated with increased tumor invasion, an advanced tumor grade, and frequent disease relapse in clinical samples of OSCC [39], suggesting CAF’s involvement in promoting OSCC malignancy. Interestingly, we found that OSCC CSCs secreted more EVs (including the cargos) than their parental counterparts. This could possibly be one of the routes through which CSCs transform NGFs into CAFs; CSCs thus create a protumor growth niche by expanding the CAF pool in the TME. CAFs have also been shown to enhance the metastatic potential of lung cancers via IL-6/STAT3 signalling pathways [40]. Importantly, studies associated with STAT3 also showed its involvement in enhancing the tumor growth and metastasis of human head and neck squamous cell carcinoma (HNSCC) [41]. In agreement, as described in Supplementary Figure S1, when STAT3 was silenced, CAL27 and SCC-15 cells lost a significant degree of ability to generate tumor spheres and the EVs released from STAT3-silenced tumor spheres, contained a lower level of β-catenin, TGFβ1 and miR-21-5p; these observations strongly suggest that inhibiting signaling networks such as STAT3/β-catenin, associated with the generation of cancer stem cells, could lead to the reduced oncogenic cargos released into the TME. 

Based on previous studies by our team, OV exhibits antibacterial, anti-inflammatory, and anticancer properties [8,9,22]. The present study provides an additional mechanistic insight into the ability of OV to suppress OSCC tumorigenesis by reducing the cargo of CSC-derived EVs. OV treatment suppressed the self-renewal capability of CAL27 and SCC-15 cells and reduced the cargo within EVs generated from CSCs, highlighting the therapeutic potential of OV, since EV cargos are considered one of the major routes for cancer cells to communicate with each other and with the TME [42,43]. It is also relevant that OV treatment significantly reduced the miR-21-5p level within CSC_EVs alongside STAT3 and mTOR, especially as evidence abounds that increased miR-21 levels in breast cancer cells are associated with inhibition of tumor suppressors, such as PDCD4 and PTEN, coupled with amplification of mTOR signaling and promotion of CAF transformation via suppression of Smad7 [18,44,45]. These findings provide additional support for our findings and the therapeutic potential of OV.

Finally, we demonstrated that OV alone or in combination with CDDP profoundly inhibited tumor growth while concurrently suppressing the tumorsphere-forming ability and decreasing EV cargos of miR-21-5p, mTOR, PI3K, STAT3, and β-catenin, indicating that OV enhances the sensitivity of CAL27 CSCs to CDDP and suppresses their tumor-initiating ability in vivo. This is consistent with previous reports that EVs enriched with oncomiRs promote drug resistance and tumor progression in different cancer types [12,46,47]. Moreover, reports have shown that EV cargos also contain signaling molecules such as phosphorylated Akt [48] and β-catenin [49], which facilitate the progression of cancer. Herein, we demonstrated that EVs from CSCs transport oncogenic cargos and promote CDDP resistance, self-renewal, and CAF transformation. The therapeutic effect of OV was associated with decreased oncogenic contents of EV cargos in OSCC cells and CSCs. While our understanding of these documented findings is still evolving, these findings provide strong support for further development of OV as a therapeutic agent for treating CDDP-resistant OSCC. A more comprehensive profiling of EV cargos isolated from OV treated OSCC tumor spheres is being investigated in our laboratory and this information will further provide insights into the signaling networks utilized by the cancer stem cells to establish a pro-tumor microenvironment.

## 5. Conclusions

In summary, our results suggested that OV treatment targets CSC_EVs, sensitizes CSCs to CDDP treatment, and suppresses OSCC tumorigenesis by reducing cargos of CSC-derived EVs (containing mTOR, PI3K, STAT3, β-catenin, and miR-21-5p) while concomitantly suppressing the self-renewal capability and CAF transformation of OSCC cells by disrupting EV communication with the TME. Our findings highlight the therapeutic efficacy of OV against therapy-resistant OSCC.

## Supplementary Materials

The following are available online at https://www.mdpi.com/2072-6694/12/1/56/s1, Figure S1: STAT3 silencing resulted in reduced OSCC tumorigenesis/stemness and oncogenic exosomal cargo., Table S1: Primary antibodies used in this study and testing conditions, Table S2: QPCR primer sequences used in this study.
