# Supplementary material for: Ovatodiolide Suppresses Oral Cancer Malignancy by Down-Regulating Exosomal Mir-21/STAT3/β-Catenin Cargo and Preventing Oncogenic Transformation of Normal Gingival Fibroblasts
Source: Cancers (Basel). 2019 Dec 24;12(1):56. doi: 10.3390/cancers12010056 (PMC7017298; doi:10.3390/cancers12010056)
Supplement: Supplementary file 1 [file cancers-12-00056-s001.zip › Authors' R3 western AW 20191211.pptx]

## Slide 1
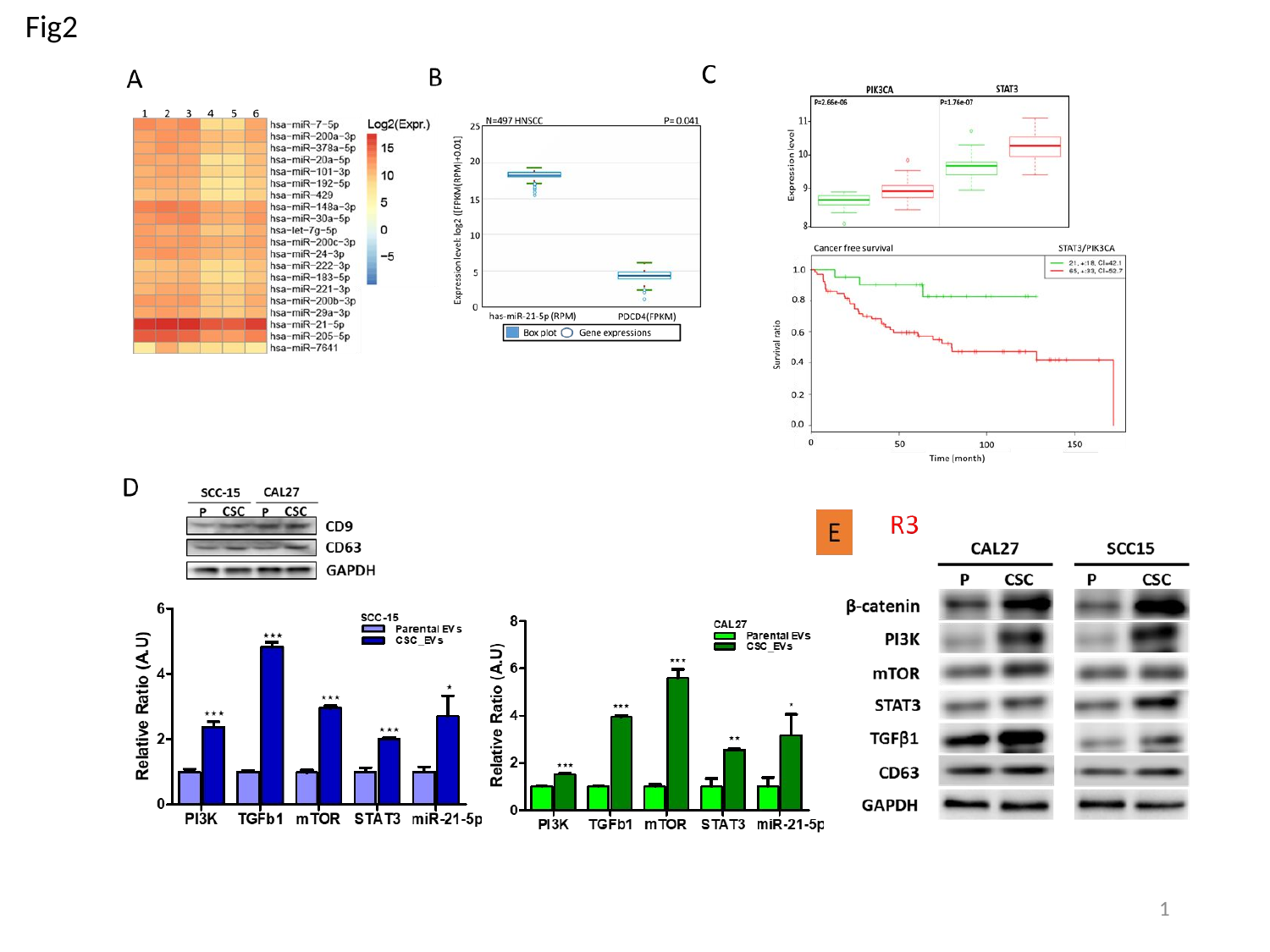

Fig2
1

## Slide 2
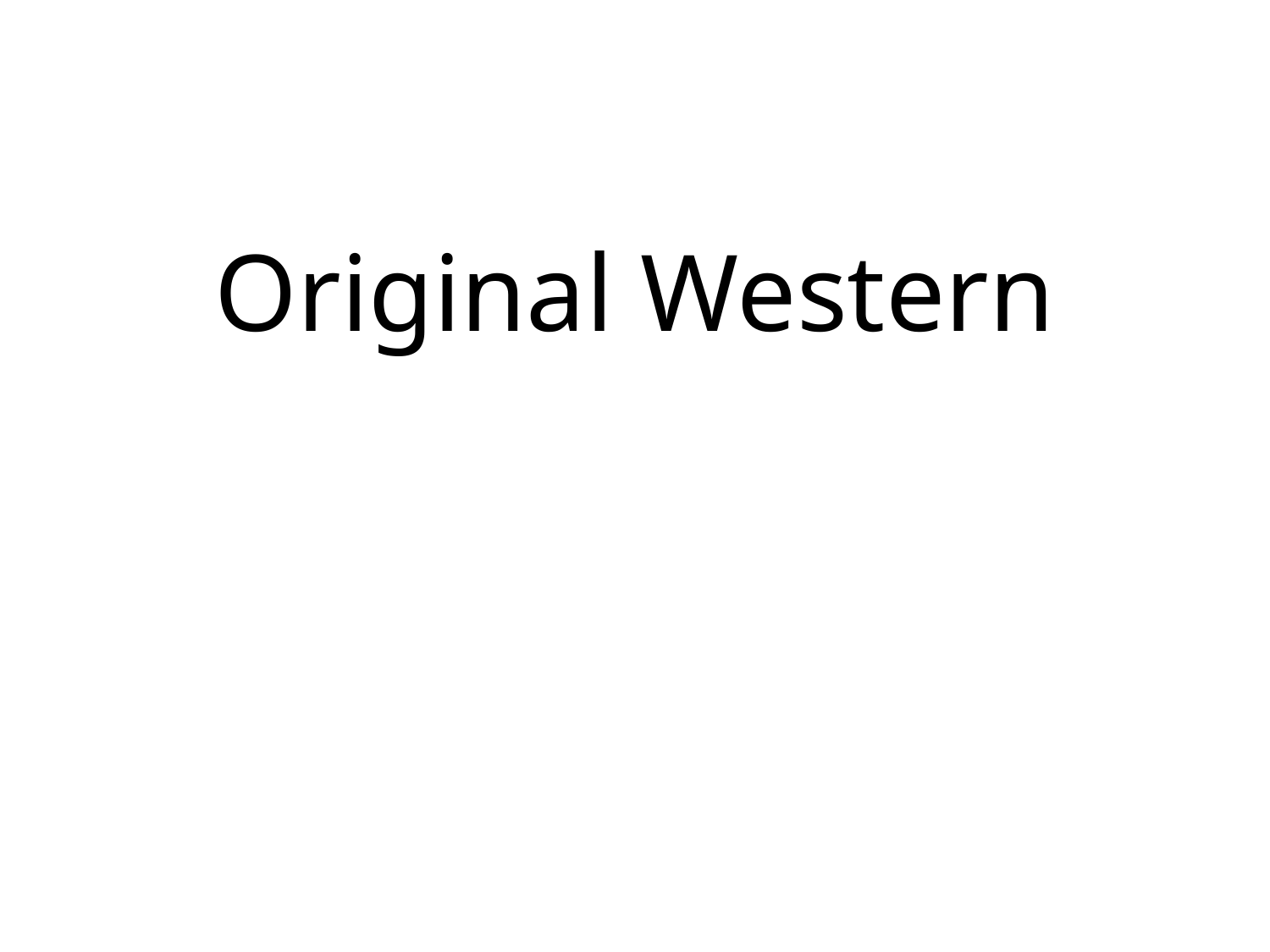

# Original Western

## Slide 3
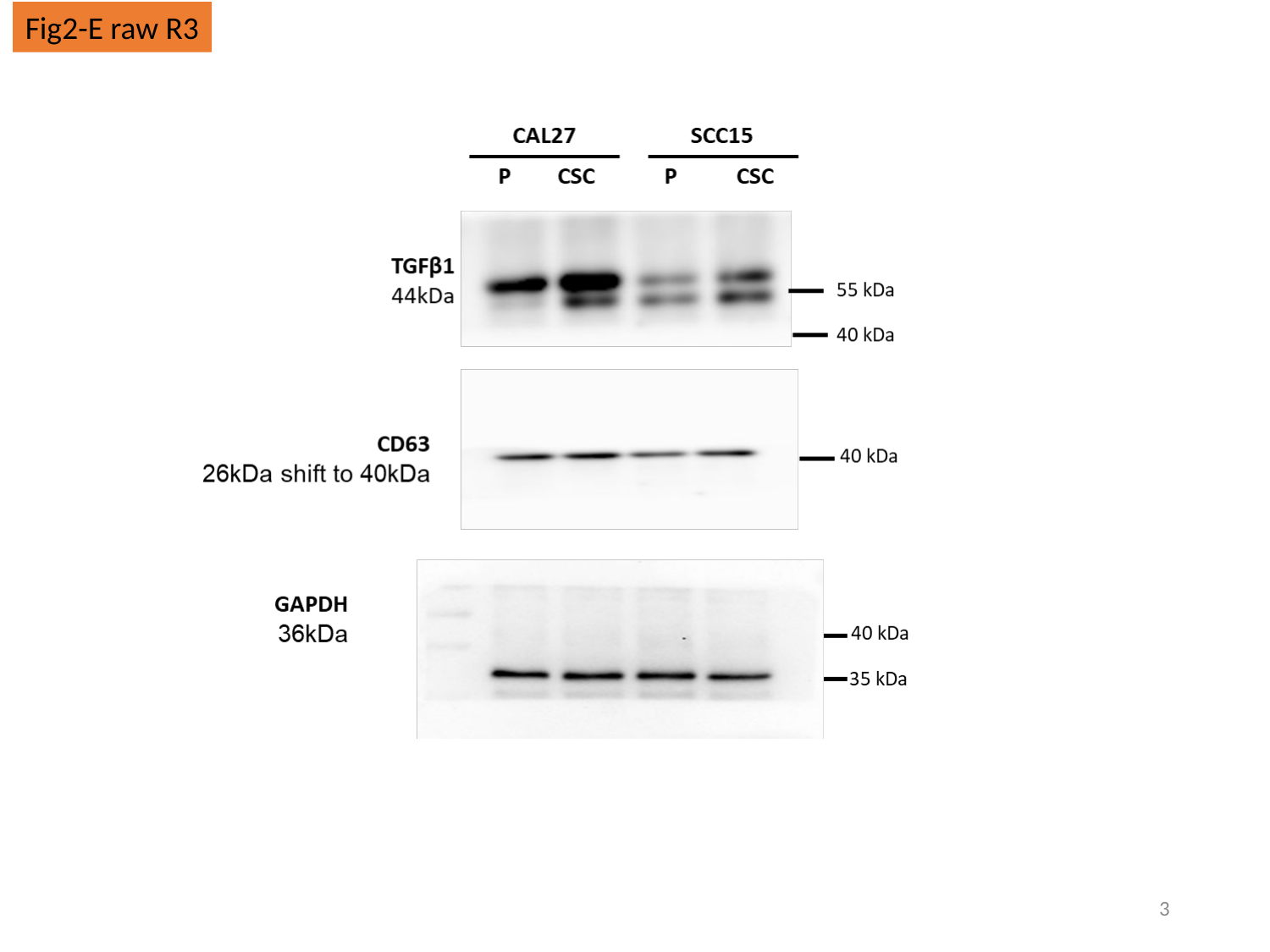

Fig2-E raw R3
3

## Slide 4
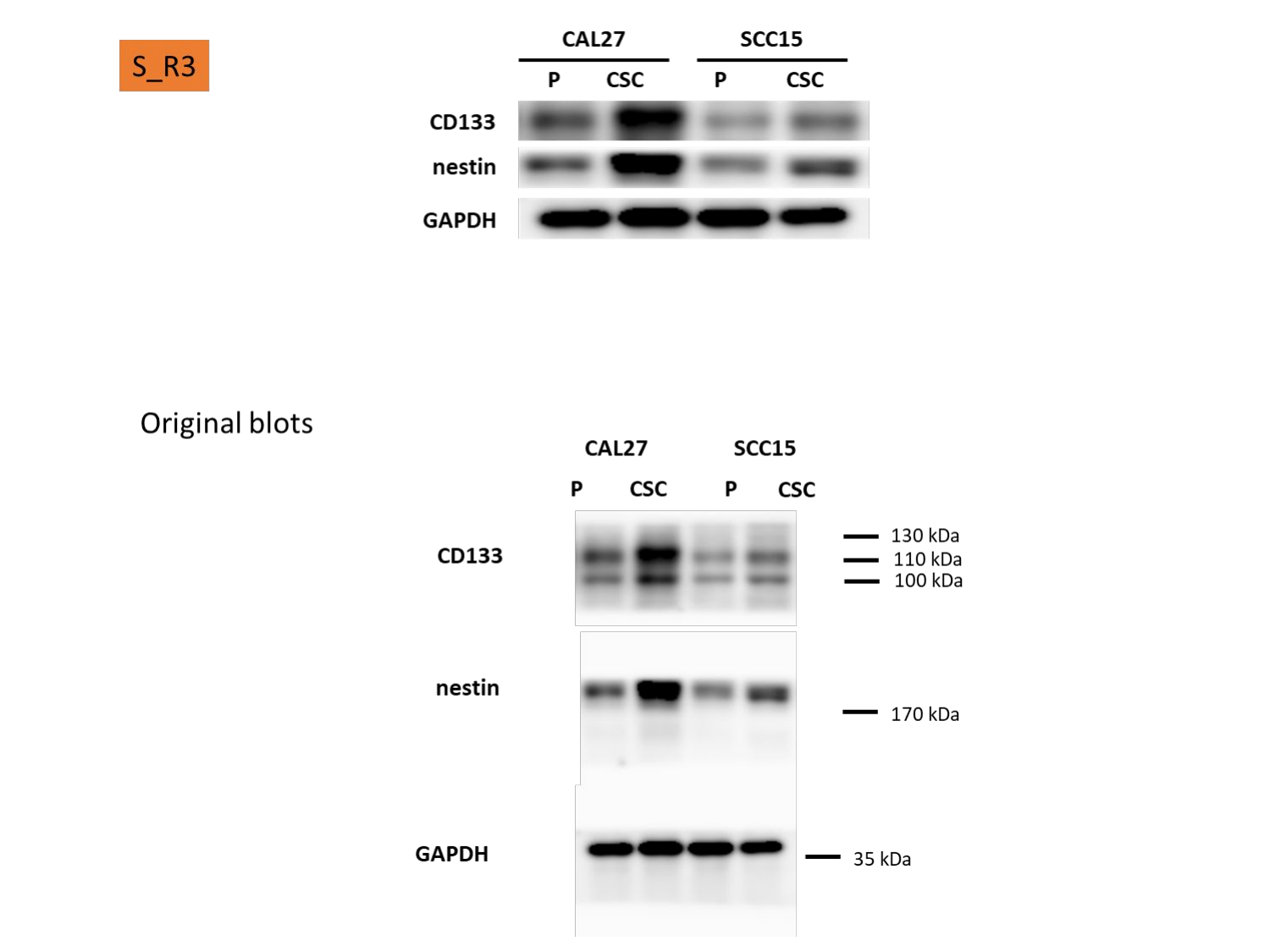

## Slide 5
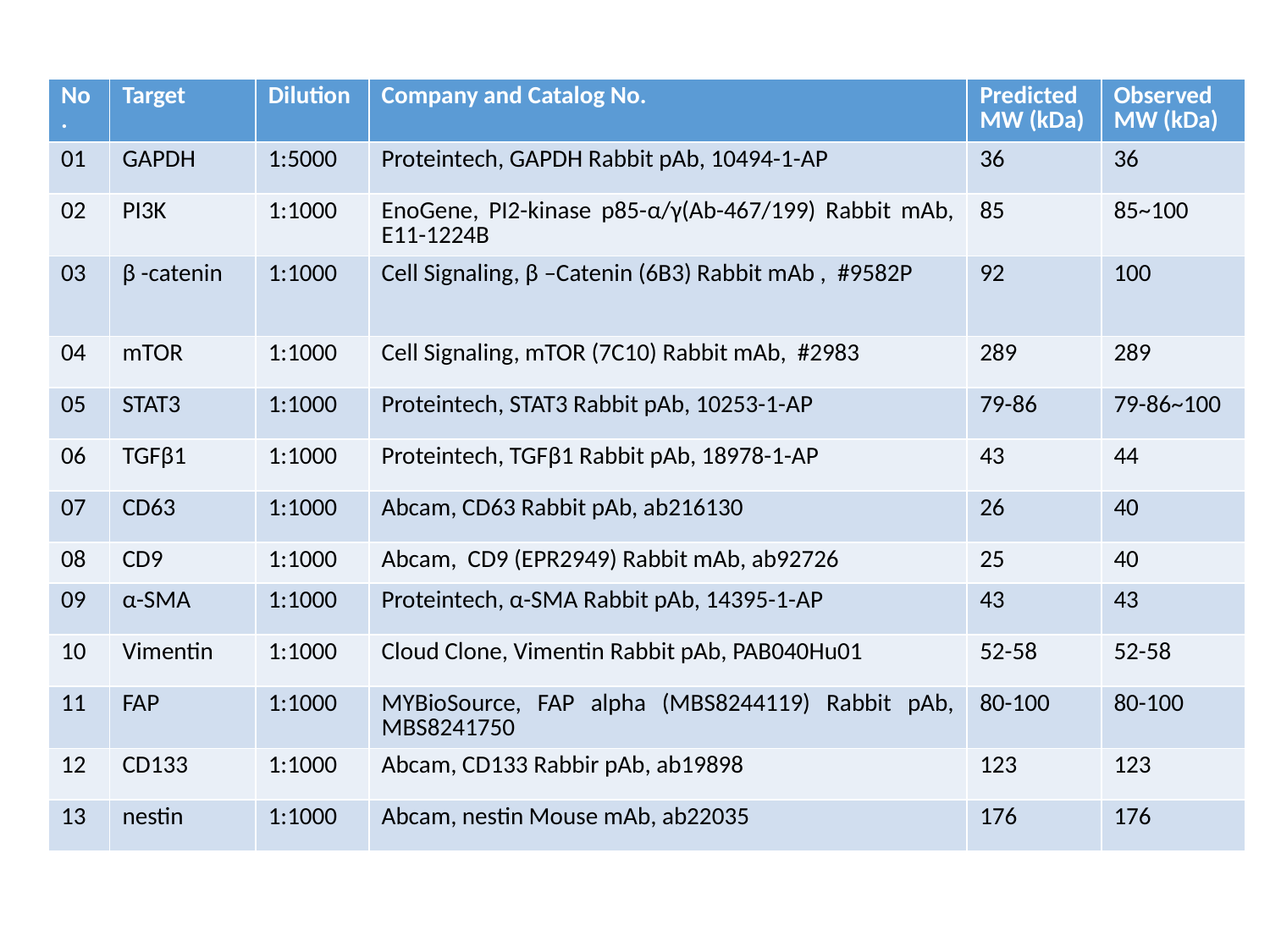

| No. | Target | Dilution | Company and Catalog No. | Predicted MW (kDa) | Observed MW (kDa) |
| --- | --- | --- | --- | --- | --- |
| 01 | GAPDH | 1:5000 | Proteintech, GAPDH Rabbit pAb, 10494-1-AP | 36 | 36 |
| 02 | PI3K | 1:1000 | EnoGene, PI2-kinase p85-α/γ(Ab-467/199) Rabbit mAb, E11-1224B | 85 | 85~100 |
| 03 | β -catenin | 1:1000 | Cell Signaling, β –Catenin (6B3) Rabbit mAb , #9582P | 92 | 100 |
| 04 | mTOR | 1:1000 | Cell Signaling, mTOR (7C10) Rabbit mAb, #2983 | 289 | 289 |
| 05 | STAT3 | 1:1000 | Proteintech, STAT3 Rabbit pAb, 10253-1-AP | 79-86 | 79-86~100 |
| 06 | TGFβ1 | 1:1000 | Proteintech, TGFβ1 Rabbit pAb, 18978-1-AP | 43 | 44 |
| 07 | CD63 | 1:1000 | Abcam, CD63 Rabbit pAb, ab216130 | 26 | 40 |
| 08 | CD9 | 1:1000 | Abcam, CD9 (EPR2949) Rabbit mAb, ab92726 | 25 | 40 |
| 09 | α-SMA | 1:1000 | Proteintech, α-SMA Rabbit pAb, 14395-1-AP | 43 | 43 |
| 10 | Vimentin | 1:1000 | Cloud Clone, Vimentin Rabbit pAb, PAB040Hu01 | 52-58 | 52-58 |
| 11 | FAP | 1:1000 | MYBioSource, FAP alpha (MBS8244119) Rabbit pAb, MBS8241750 | 80-100 | 80-100 |
| 12 | CD133 | 1:1000 | Abcam, CD133 Rabbir pAb, ab19898 | 123 | 123 |
| 13 | nestin | 1:1000 | Abcam, nestin Mouse mAb, ab22035 | 176 | 176 |
