# Supplementary material for: Ovatodiolide Suppresses Oral Cancer Malignancy by Down-Regulating Exosomal Mir-21/STAT3/β-Catenin Cargo and Preventing Oncogenic Transformation of Normal Gingival Fibroblasts
Source: Cancers (Basel). 2019 Dec 24;12(1):56. doi: 10.3390/cancers12010056 (PMC7017298; doi:10.3390/cancers12010056)
Supplement: Supplementary file 1 [file cancers-12-00056-s001.zip › supplementary figure1.pdf]

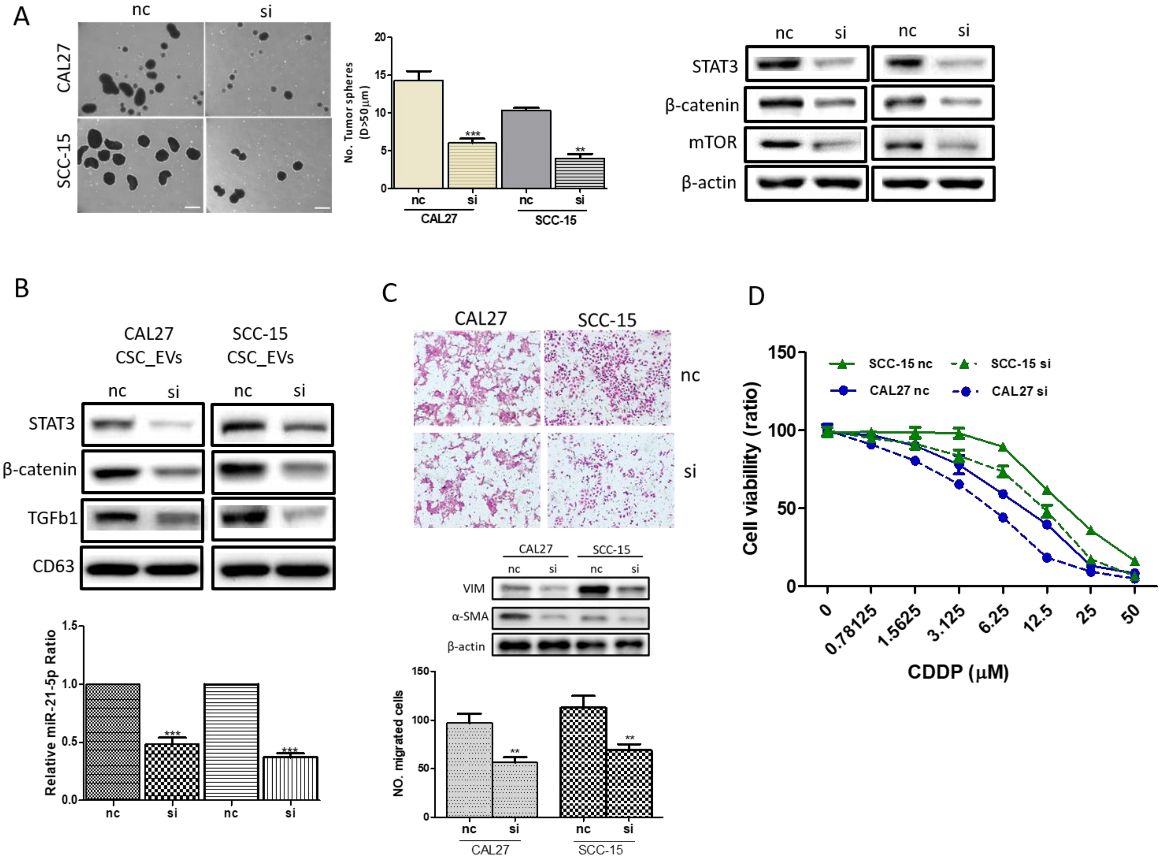

Supplementary Figure 1. STAT3 silencing resulted in reduced OSCC tumorigenesis/stemness and oncogenic exosomal cargo. (A) Left, representative micrographs of tumor spheres. Tumor sphere formation assay showed that silencing STAT3 (si) in CAL27 and SCC-15 resulted in a significantly reduced tumor sphere forming ability as compared to their counterparts (nc, negative control). Middle, comparative bar graph of tumor sphere formation. Right, western blots of both STAT3-silenced (si) CAL27 and SCC-15 cells as compared to their control (nc) counterparts. (B) Western blots of EVs isolated from control (nc) and STAT3-silenced (si) OSCC cells. CD63 was used as loading control and marker of EVs. A significantly lower miR-21-5p level in EVs isolated in STAT3-silenced tumor spheres of CAL27 and SCC-15. (C) Migration assay of CAFs transformed by EVs collected from control (nc) OSCC spheres and EVs from STAT3-silenced (si) counterparts. Insert indicates the Western blots showing reduced markers for CAFs, VIM (vimentin) and α-SMA in CAFs transformed by EVs from tumor spheres generated from STAT3-silenced OSCC cells. (D) Cell viability assay comparing CDDP responses in OSCC cells treated with EVs isolated from control tumor spheres (nc) and EVs from tumor spheres with STAT3-silenced. **$p<0.01$; ***$p<0.001$.
