# Supplementary material for: Ovatodiolide Suppresses Oral Cancer Malignancy by Down-Regulating Exosomal Mir-21/STAT3/β-Catenin Cargo and Preventing Oncogenic Transformation of Normal Gingival Fibroblasts
Source: Cancers (Basel). 2019 Dec 24;12(1):56. doi: 10.3390/cancers12010056 (PMC7017298; doi:10.3390/cancers12010056)
Supplement: Supplementary file 1 [file cancers-12-00056-s001.zip › Supplementary tables.pdf]

Supplementary Table 1. Primary antibodies used in this study and testing conditions

No.	Target	Dilution	Company and Catalog No.	Predicted MW (kDa)	Observed MW (kDa)
01	GAPDH	1:5000	Proteintech, GAPDH Rabbit pAb, 10494-1-AP	36	36
02	PI3K	1:1000	EnoGene, PI2-kinase p85-α/γ(Ab-467/199) Rabbit mAb, E11-1224B	85	85~100
03	β-catenin	1:1000	Cell Signaling, β –Catenin (6B3) Rabbit mAb , #9582P	92	100
04	mTOR	1:1000	Cell Signaling, mTOR (7C10) Rabbit mAb, #2983	289	289
05	STAT3	1:1000	Proteintech, STAT3 Rabbit pAb, 10253-1-AP	79-86	79-86~100
06	TGFβ1	1:1000	Proteintech, TGFβ1 Rabbit pAb, 18978-1-AP	43	44
07	CD63	1:1000	Abcam, CD63 Rabbit pAb, ab216130	26	40
08	CD9	1:1000	Abcam, CD9 (EPR2949) Rabbit mAb, ab92726	25	40
09	α-SMA	1:1000	Proteintech, α-SMA Rabbit pAb, 14395-1-AP	43	43
10	Vimentin	1:1000	Cloud Clone, Vimentin Rabbit pAb, PAB040Hu01	52-58	52-58
11	FAP	1:1000	MYBioSource, FAP alpha (MBS8244119) Rabbit pAb, MBS8241750	80-100	80-100
12	CD133	1:1000	Abcam, CD133 Rabbit pAb, ab19898	123	123
13	nestin	1:1000	Abcam, nestin Mouse mAb, ab22035	176	176

Supplementary Table 2. QPCR primer sequences used in this

mRNA	Forward sequence	Reverse sequence
**PI3K**	AGTAGGCAACCGTGAAGAAAAG	GAGGTGAATTGAGGTCCCTAAGA
**TGFb1**	CACCCGCGTGCTAATGG	TGTGTACTCTGCTTGAACTTGTCAT
**mTOR**	CTGGGACTCAAATGTGTGCAGTTC	GAACAATAGGGTGAATGATCCGGG
**STAT3**	ACCAGCAGTATAGCCGCTTC	GCCACAATCCGGGCAATCT
**Akt**	TCACCTCTGAGACCGACACC	ACTGGCTGAGTAGGAGAACTGG
**GAPDH**	CATCATCCCTGCCTCTACTG	GCCTGCTTCACCACCTTC
